# Can late stage marine mortality explain observed shifts in age structure of Chinook salmon?

**DOI:** 10.1371/journal.pone.0247370

**Published:** 2021-02-19

**Authors:** Kaitlyn A. Manishin, Curry J. Cunningham, Peter A. H. Westley, Andrew C. Seitz

**Affiliations:** 1 College of Fisheries and Ocean Sciences, University of Alaska Fairbanks, Fairbanks, Alaska, United States of America; 2 NOAA, Juneau, Alaska, United States of America; Texas A&M University, UNITED STATES

## Abstract

Chinook salmon (*Oncorhynchus tshawytscha*) populations have experienced widespread declines in abundance and abrupt shifts toward younger and smaller adults returning to spawn in rivers. The causal agents underpinning these shifts are largely unknown. Here we investigate the potential role of late-stage marine mortality, defined as occurring after the first winter at sea, in driving this species’ changing age structure. Simulations using a stage-based life cycle model that included additional mortality during after the first winter at sea better reflected observed changes in the age structure of a well-studied and representative population of Chinook salmon from the Yukon River drainage, compared with a model estimating environmentally-driven variation in age-specific survival alone. Although the specific agents of late-stage mortality are not known, our finding is consistent with work reporting predation by salmon sharks (*Lamna ditropis*) and marine mammals including killer whales (*Orcinus orca*). Taken as a whole, this work suggests that Pacific salmon mortality after the first winter at sea is likely to be higher than previously thought and highlights the need to investigate selective sources of mortality, such as predation, as major contributors to rapidly changing age structure of spawning adult Chinook salmon.

## Introduction

Changes in age structure have been well documented in exploited species such as fish populations experiencing top-down pressure from fisheries or natural predators [1]. The ecological consequences of age structure changes, such as reduced egg and offspring quality, have been hypothesized to reduce productivity of populations, which is concerning for imperiled species that are failing to recover despite reduction in fishing mortality or the implementation of fishery closures [2, 3]. Evidence that demographic changes can be the result of top-down control has also been revealed from controlled experiments [4]. For example, guppy (*Poecilia reticulata*) populations that live in high predation environments display age structure truncation [5]. Therefore, identifying the causes of demographic changes is a crucial step toward understanding the lack of recovery by imperiled fish stocks [1, 6].

Chinook salmon (*Oncorhynchus tshawytscha*) is an anadromous and semelparous species, and the largest-bodied of the Pacific salmonids. Marked variation among populations notwithstanding, adult Chinook salmon typically spawn in rivers during the summer or fall, after which the embryos hatch during the winter and emerge as free-swimming individuals the following spring. In the northern portion of their range, the young typically spend one full year in freshwater before transitioning to smolts and migrating to sea. The majority of lifetime growth of Chinook salmon occurs in the North Pacific Ocean. Chinook salmon spend between two and five years feeding on both invertebrate and fish prey, before returning to natal river systems for reproduction and death. Chinook salmon have decreased in abundance, and size- and age-at-maturity throughout the Pacific basin, though the magnitude of the trends varies regionally [7, 8]. Probability of maturity in salmonids is thought to be related to size-at-age, such that, smaller size-at-age is predicted to result in increased age-at-maturity [9, 10]. In Chinook salmon, both size-at-age and age-at-maturity have decreased [11], the opposite of what is expected if maturation is simply plastic with respect to growth [7, 8]. The decrease in age-at-maturity has resulted in the truncation of the older component of the age structure in many populations. The most pronounced shifts in age structure have occurred in Alaska, the northern-most part of the species’ range [8], where the oldest returning spawners (ocean-age 5 and older) have become very rare or have disappeared altogether. The loss of older adults has direct ecological and demographic consequences for Chinook salmon populations, and economic and social consequences for the humans that rely on this resource.

The basin-wide extent of this age structure change suggests exposure to shared marine drivers is likely responsible. Currently, it is widely assumed that the risk of mortality decreases dramatically after the first winter in the ocean, the marine environment is relatively safe thereafter, and that effects in this ‘late’ marine stage have minimal impacts on population characteristics, including age structure [12]. An alternative hypothesis to explain the changes observed in Chinook salmon age structure is that late-stage mortality, defined hereafter as occurring after the first winter at-sea, is common [11]. Under this hypothesis, the period of Chinook salmon life history thought to define population characteristics is expanded beyond the known critical periods of ocean entry and the first ocean winter [11].

Here we investigate the importance of late-stage marine mortality in driving changes in age structure of Chinook salmon by examining at which ocean ages mortality that was unaccounted for in previous research must be applied to replicate observed changes in age structure of a representative Chinook salmon population in the Yukon River watershed [13]. Specifically, we use simulation analysis to test the hypothesis that frequent late-stage marine mortality could better explain observed patterns in the age structure of return adult salmon at maturation, compared with the influence of environmentally-driven survival variation alone. To simulate the outcomes of several scenarios for late-stage marine mortality, we utilized results from a stage-structured estimation model parameterized to the life cycle of Yukon River Chinook salmon [13] that controls for known natural and anthropogenic drivers of variation in survival across life-history stages, including environmental factors, and both directed and incidental fisheries removals. The unamended model systematically under-represented the proportion of ocean-age 2 returns and over-represented the proportion of ocean-age 3 adults returning to the Salcha River [13]. Here, we investigate under what combination of intensity and ocean-age selectivity could marine mortality be added to the original model to more accurately replicate observed patterns in adult age composition.

## Methods

### Study system and design

We simulated the impact of late-stage marine mortality on the spawning population age structure of Chinook salmon from the Salcha River, a tributary to the Yukon River, under alternative scenarios describing the intensity and selectivity of additional mortality in the ocean after the first winter at-sea. The Salcha River represents an optimal indicator for the Yukon River watershed as it is the largest single producer of Chinook salmon in the drainage [14]. Additionally, this population has a breadth of data available and has been the focus of previous evaluations of Yukon River Chinook salmon population dynamics, including the life-cycle model [13] upon which we base our simulations. This life-cycle estimation model attempted to explain past variation in age-specific Chinook salmon abundance between 1987 and 2010 [13, 15] based on a suite of natural and anthropogenic factors, by fitting a broad array of abundance, age composition, catch, and survey information.

**Fig 1:**
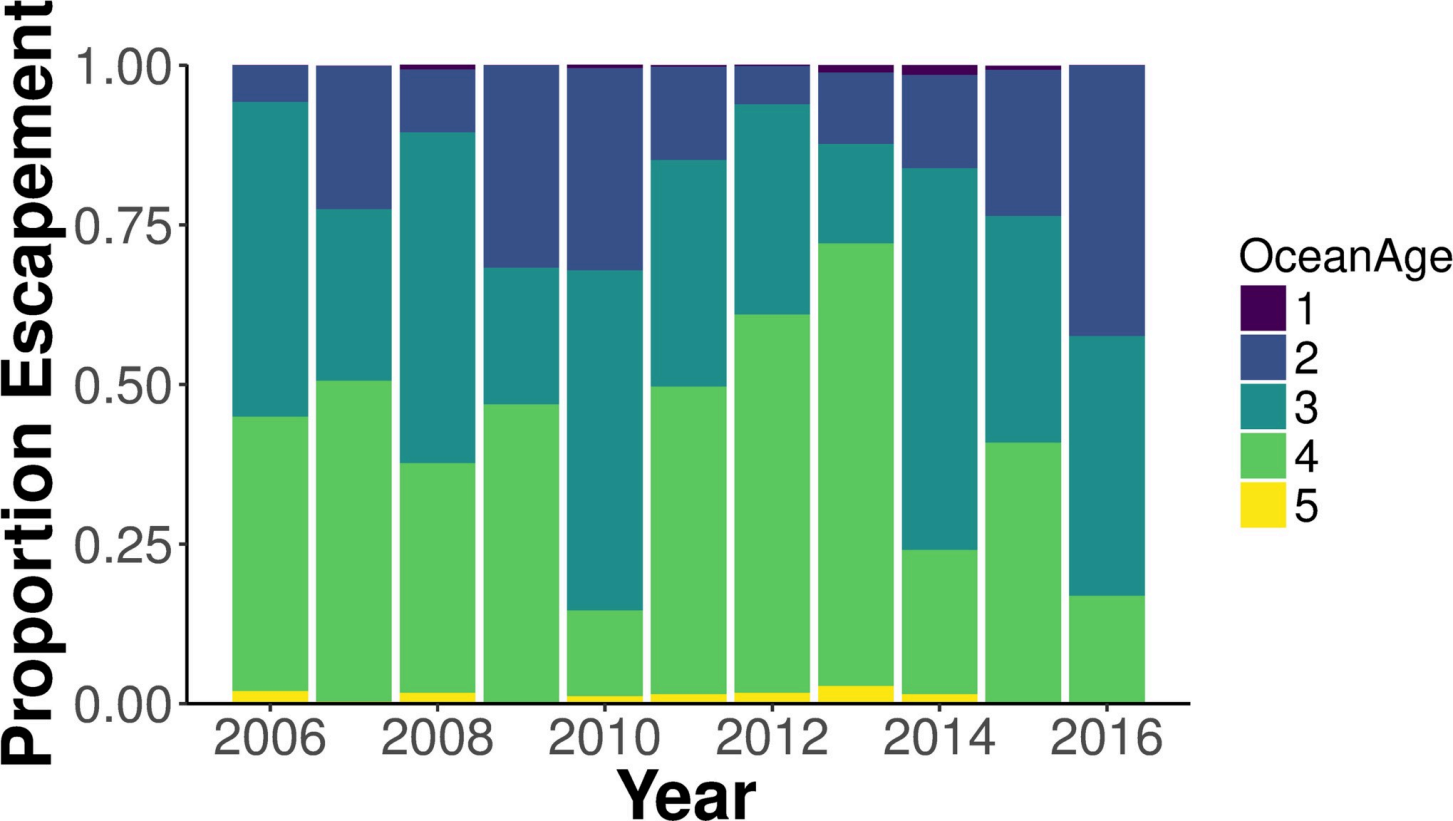
The age composition of Chinook salmon adult returns to the Salcha River, 2006–2016 [16–26].

To understand changes in the age structure at a finer temporal scale than previous research (e.g., [7]), we examined the age structure of adult returns to the Salcha River from 2006 to 2016 (Fig 1 and S1 File) as reported by the Joint Technical Committee of the U.S./Canada Yukon River Panel [16–26]. The time series from the Joint Technical Committee and life cycle model [13] differ in length, but 2006 through 2010 are included in both. Thus, the spawning age composition from years 2006 to 2010 was used as a comparison period with simulations based on the life-cycle model.

### Simulation model structure

Our simulation framework was built upon a previous Bayesian stage-structured life-cycle model that tracks cohorts of Chinook salmon from specific brood years (i.e., all the individuals whose life cycle started as eggs in the same year) through sequential life stages culminating in spawning adults returning to the Salcha River and is fully described by [13]. In brief, the estimated maturation schedule, stage-specific survival rates and capacities, and age-specific fecundities estimated by the stage-structured population dynamics model fit to this population, along with the uncertainty in those estimated model parameters, were used to parameterize our simulations [13]. Parameter values for the simulations were drawn from the joint posterior distribution of the fitted model to propagate estimation uncertainty through simulations while maintaining the correlation structure among model parameters. Cunningham et al. [13] fit this model accounting for the environmental and anthropogenic factors. The model was used as the base operating model for the simulation analysis described in this paper; we performed no additional fitting.

Survival (*SR*_*y*,*s*_) of a Chinook salmon cohort from a specific brood year (*y*) through a specific life stage (*s*) was approximated by a Beverton-Holt transition function [27]:

SRy,s=py,s1+py,sNy,s−1ks, (1)

where *N* was abundance from that brood year (*y*) during the previous life stage (*s-1*). Productivity (*p*_*y*,*s*_) was the maximum survival rate to the next life stage and was represented by a linear time-varying function of average productivity [13] and environmental covariates. Capacity (*k*_*s*_) was the total rearing potential of that stage; one capacity parameter was estimated for all freshwater stages and another for all marine stages. Capacities were assumed constant among brood years. Basal productivity, or average survival before the additive effects of environmental covariates, was estimated by Cunningham et al. [13] to increase linearly (in logit space) across ocean stages. Model stages were associated with distinct periods in the life cycle and could be directly related to ocean ages, or the number of years a cohort of salmon had spent in the ocean.

The environmental drivers of Chinook salmon survival found by Cunningham et al. [13] to be most strongly supported were ice-out date on the Yukon River, winter temperature in the Pribilof Islands, and Japanese hatchery chum abundance. Environmental covariates were included or excluded at each stage using Bayesian model selection methods. However, the model systematically under represented the proportion of ocean-age 2 returns and over represented the proportion of ocean-age 3 adults returning to the Salcha River [13]. For additional details on the estimation model used as the basis for simulations, see Cunningham et al. [13].

### Simulation design

To simulate additional mortality and identify under what combination of selectivity and intensity this additional mortality can best describe observed trends in adult Chinook salmon age structure, we added a term representing ocean-age specific instantaneous mortality (*λ*_*a*_) to several of the state equations [13]. Chinook salmon abundance was represented by *N*_*y*, *s*_ or the number of individuals from brood year y, surviving to the end of stage s,

$$N_{y,s}=\left(N_{y,s-1}\right)e^{-(S_{a}\cdot F_{t}+\lambda_{a}+M_{y,s})}\left(1-\phi_{a}\right).$$ (2)

Where the additional instantaneous mortality rate for Chinook salmon during their *a*-th year in the ocean is *λ*_*a*_, *S*_*a*_ is bycatch selectivity by ocean age *a*, *F*_*t*_ is the annual bycatch fishing mortality rate in year *t*, and *ϕ*_*a*_ is the ocean-age specific probability of maturing. Maturation probabilities at ocean age (*ϕ*_*a*_) were assumed to follow a logistic function, the parameters of which were directly estimated from the data. Calendar year, *t*, is offset from brood year, *y*, based on the timing of each marine stage, and likewise ocean age *a* was appropriately indexed to each model stage *s*, as *a* = *s*– 2.

Additional mortality was also included in calculations for the number of Chinook salmon surviving to return to freshwater spawning grounds [13]:

$$R_{t,a}=\left(N_{y,s-1}\right)e^{-(S_{a}\cdot F_{t}+\lambda_{a}+M_{y,s})}(\phi_{a})(1-U_{t}).$$ (3)

The number of individuals returning to spawn, *R*_*t*,*a*_, of ocean age *a* in calendar year *t*, is the number of surviving individuals, less those caught in terminal commercial, recreational, and subsistence fisheries where *U*_t_ is the observed annual harvest rate.

The number of fish removed by additional mortality from each ocean age class and year was calculated using a modified Baranov catch equation [28], with our additional mortality substituted for catch:

$$P_{t,a}=\left(\frac{\lambda_{a}}{S_{a}\cdot F_{t}+\lambda_{a}+M_{y,s}}\right)\cdot \left(N_{y,s-1}\right)\cdot \left(1-e^{-(S_{a}\cdot F_{t}+\lambda_{a}+M_{y,s})}\right).$$ (4)

Additional late-stage mortality by year and ocean age are given by *P*_*t*,*a*_. The first term on the right side of Eq 4 is the proportion of total mortality (denominator) due to the additional mortality explored in simulations (numerator), the second term is number of fish entering that stage, and the final (third) term describes the overall mortality rate.

A range of values for *λ*_*a*_, describing additional age-specific marine mortality, were proposed and used to simulate Chinook salmon abundance in return years 1987 through 2010. In each year of the simulation, spawning abundance and proportion of females on the spawning grounds in the previous year were used to calculate total egg deposition. Fish advanced from one model stage to the next depending on the survival rate described by Eq 1. For each ocean stage in a given calendar year, the number of fish removed from the population due to the additional mortality, returning to the river, escaping terminal freshwater harvest, and predicted to arrive on the spawning grounds was recorded. The annual age composition of adult spawning salmon was calculated by dividing the return abundance of each age class in a given calendar year, by the sum of all returning salmon in that year. The simulation was repeated 100 times for each value of *λ*_*a*_ with random draws for demographic parameters from the joint posterior for parameters as estimated in Cunningham et al. [13].

### Marine mortality scenarios

Scenarios (*λ*_*a*_) describe the additional instantaneous late-stage mortality imposed on Chinook salmon of each ocean age (i.e. ocean-ages 1 through 5). Additional mortality was apportioned among age classes using a vector of proportions termed a “selectivity suite” (*q*_*a*_), which was then multiplied by a scalar that quantified the total intensity (*I*) of simulated mortality. Each selectivity suite summed to one so the value of a given age can be viewed as the proportion of additional mortality allocated to that age class.

$$\lambda_{a}=I\cdot q_{a},\;\text{where}\;\alpha =1:5.$$ (5)

Scenarios were identified by their selectivity suite number, a dash, and the overall intensity. For example, scenario “1–0.5” was selectivity suite 1 multiplied by an instantaneous mortality rate of *I* = 0.5. Because the additional mortality resulted in varying numbers of fish removed from the population depending on the number of fish in each ocean age class, we tracked the additional fish removed in a calendar year and calculated percentage of fish removed and total additional realized mortality rate post hoc.

Alternative scenarios that varied in both intensity and allocation of that mortality across age classes (selectivity) were explored iteratively. All selectivity suites were simulated with total intensities of *I* = 0–2, in increments of 0.2, meaning that the minimum additional instantaneous mortality rate in a given age class is 0 and the maximum is 2. An intensity of 0 adds no additional mortality and allows for comparison to the baseline lifecycle model. All selectivity suites, prior to scaling by total intensity, are compared in Table 1.

**Table 1:** Selectivity suites (*q*_*a*_) determine how additional late-stage mortality is apportioned among ocean age classes (*a*), which are then scaled by different intensities (*I*) of 0–2.0 to form mortality scenarios (*λ*_*a*_).

**Selectivity Suite (*q*)**		**Selectivity (*q***_***a***_)				
		**Ocean Ages**				
		**1**	**2**	**3**	**4**	**5**
	**1**	0.20	0.20	0.20	0.20	0.20
	**2**	0.00	0.05	0.05	0.45	0.45
	**3**	0.07	0.13	0.20	0.27	0.33
	**4**	0.33	0.27	0.20	0.13	0.07
	**5**	0.04	0.04	0.43	0.43	0.04
	**6**	0.03	0.10	0.17	0.34	0.34
	**7**	0.00	0.08	0.42	0.42	0.08
	**8**	0.00	0.16	0.26	0.05	0.53
	**9**	0.00	0.00	0.91	0.09	0.00
	**10**	0.00	0.00	1.00	0.00	0.00
	**11**	0.00	0.00	0.90	0.10	0.00
	**12**	0.00	0.00	0.80	0.20	0.00
	**13**	0.00	0.00	0.70	0.30	0.00

Instantaneous mortality rates were converted to annual percent mortality rates, while accounting for the fact that additional late-stage mortality takes place simultaneously with fishing mortality and the natural mortality predicted by environmental covariates. Therefore, to understand the effect of the additional late-stage mortality in all scenarios, we calculated the ratio of additional mortality to total marine mortality at a given age and year (Eq 6).

$$AM_{y,a}\left(\%\right)=\frac{\lambda_{a}}{\lambda_{a}+S_{a}\cdot F_{t}+M_{y,s}}\cdot \left(1-e^{-(\lambda_{a}+S_{a}\cdot F_{t}+M_{y,s})}\right)$$ (6)

The median additional mortality rate was taken over the simulation period to facilitate the comparison of scenarios. The minimum non-zero and maximum additional mortality rate for each ocean age and selectivity suite are found in Table 2.

**Table 2:** The range of annual mortality rates (%) produced by all scenarios, excluding those which added zero additional mortality when environmentally driven and bycatch mortality are accounted for. Selectivity suites (*q*_*a*_) determine how additional late-stage mortality is applied to each ocean age class, and the range describes the simulated mortality across values of different intensities (*I*).

**Selectivity Suites (*q*)**		**Additional Mortality Rate (*AM*, %)**				
		**Ocean Ages**				
		**1**	**2**	**3**	**4**	**5**
	1	0.03–0.21	0.03–0.25	0.03–0.28	0.03–0.29	0.04–0.31
	2	0.00	0.01–0.07	0.01–0.07	0.08–0.54	0.08–0.56
	3	0.01–0.08	0.02–0.18	0.03–0.28	0.05–0.37	0.06–0.45
	4	0.04–0.33	0.04–0.32	0.03–0.28	0.02–0.21	0.01–0.12
	5	0.01–0.05	0.01–0.06	0.07–0.49	0.07–0.52	0.01–0.08
	6	0.01–0.04	0.02–0.14	0.03–0.24	0.06–0.45	0.06–0.46
	7	0.00	0.01–0.12	0.07–0.48	0.07–0.51	0.02–0.14
	8	0.00	0.02–0.21	0.04–0.34	0.01–0.09	0.09–0.61
	9	0.00	0.00	0.14–0.73	0.02–0.15	0.00
	10	0.00	0.00	0.15–0.75	0.00	0.00
	11	0.00	0.00	0.14–0.73	0.02–0.16	0.00
	12	0.00	0.00	0.12–0.69	0.03–0.29	0.00
	13	0.00	0.00	0.11–0.65	0.05–0.40	0.00

The combination of selectivity suites and intensities resulted in a total of 143 scenarios of late-stage marine mortality for Chinook salmon, each of which were simulated independently. We calculated the log-likelihood of the simulated return age structure from 2006 until 2010, given the observed age composition in those years to assess their similarity. The likelihood was given by (Eq 7):

$$L(p_{y,a}|n_{y},z_{y,a})=\left(\begin{array}{c} n_{y}\\ z_{y,a} \end{array}\right)\prod_{a=2}^{4}p_{y,a}^{z_{y,a}}$$ (7)

where *n*_*y*_ is the true sample size used to generate the observed age structure from year *y* (n = 509, 308, 303, 458, 419 for 2006 through 2010, respectively [16–20]), *p*_*y*,*a*_ is the proportion of adult salmon returning at ocean age *a* in year *y* predicted from simulations, and *z*_*y*,*i*_ is the observed count of adult salmon of ocean age class *a* returning in year *y*. We initially included rarely observed ocean-age 1 and 5 returns in the scenarios, but their near zero proportion had a strong influence on the likelihood calculations while not impacting the overall age structure. To eliminate this statistical artifact, we removed these rare ages from likelihood calculations. As such, we quantified the likelihood of each marine mortality scenario, given the annual age composition data for the predominant age classes observed for Yukon River Chinook salmon. The sum of the log-likelihoods for the comparison time series (2006 through 2010) was computed to calculate the overall log-likelihood of a given mortality scenario. Additionally, because the intensity of late-stage mortality is highly uncertain, we calculated the total log-likelihood of each selectivity suite across all levels of intensity by summing the log-likelihood of each scenario from a given selectivity suite. By doing so, we were able to assess which selectivity suite was most likely to represent the observed age composition while assuming nothing about the total intensity.

The simulated total number of returning adults under the leading scenario was plotted against the original estimation model [13] to qualitatively compare the effect of the scenario on overall population abundance. We noted if the spawning adult abundance generated in simulation scenarios was within the 95% Bayesian credible interval of the spawning adult abundance from the original fitted model. Finally, to test whether inter-annual variation in the age composition of returning adults could be obscuring changes over time, we compared the simulated and observed time-series using the same methods after reducing them to smoothed trends using multinomial regression [29] (S2 File).

Ethics statement: Because we relied solely on data previously collected by other organizations for simulations in this study, no permits or animal care assurances were required.

## Results

**Fig 2:**
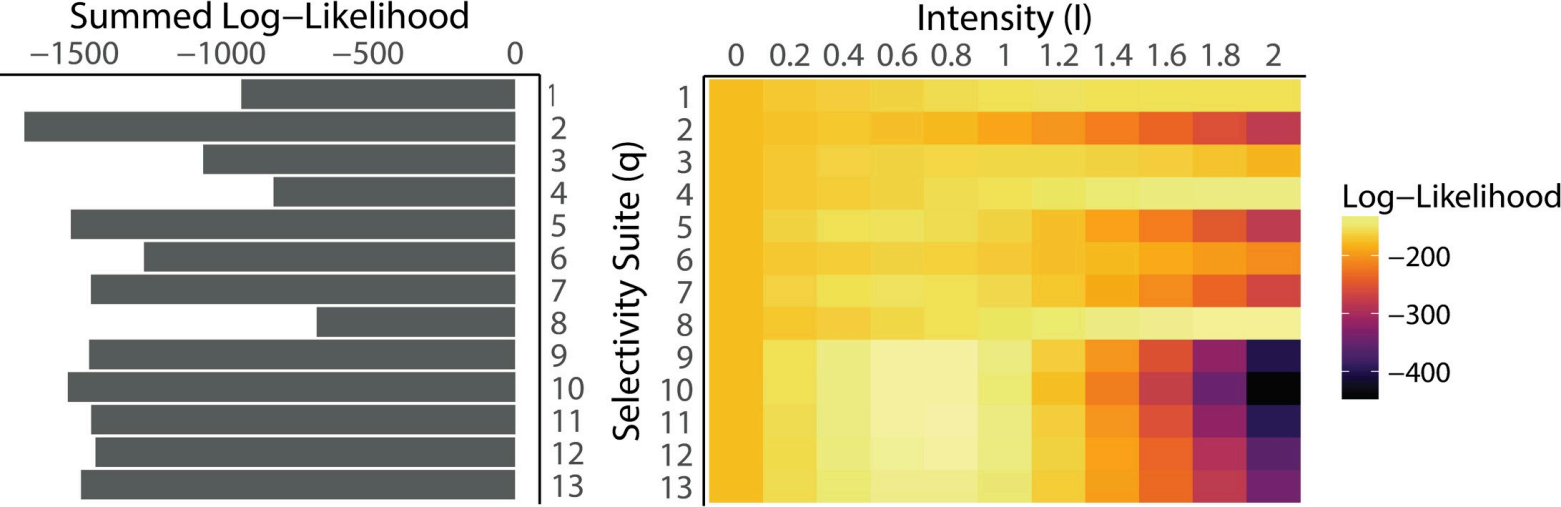
(Left Panel) Total likelihood for each selectivity suite summed over all mortality intensities. (Right Panel) Heat map of the log-likelihoods of all mortality scenarios by selectivity suite (*q*_*a*_) and overall intensity (*I*).

**Fig 3:**
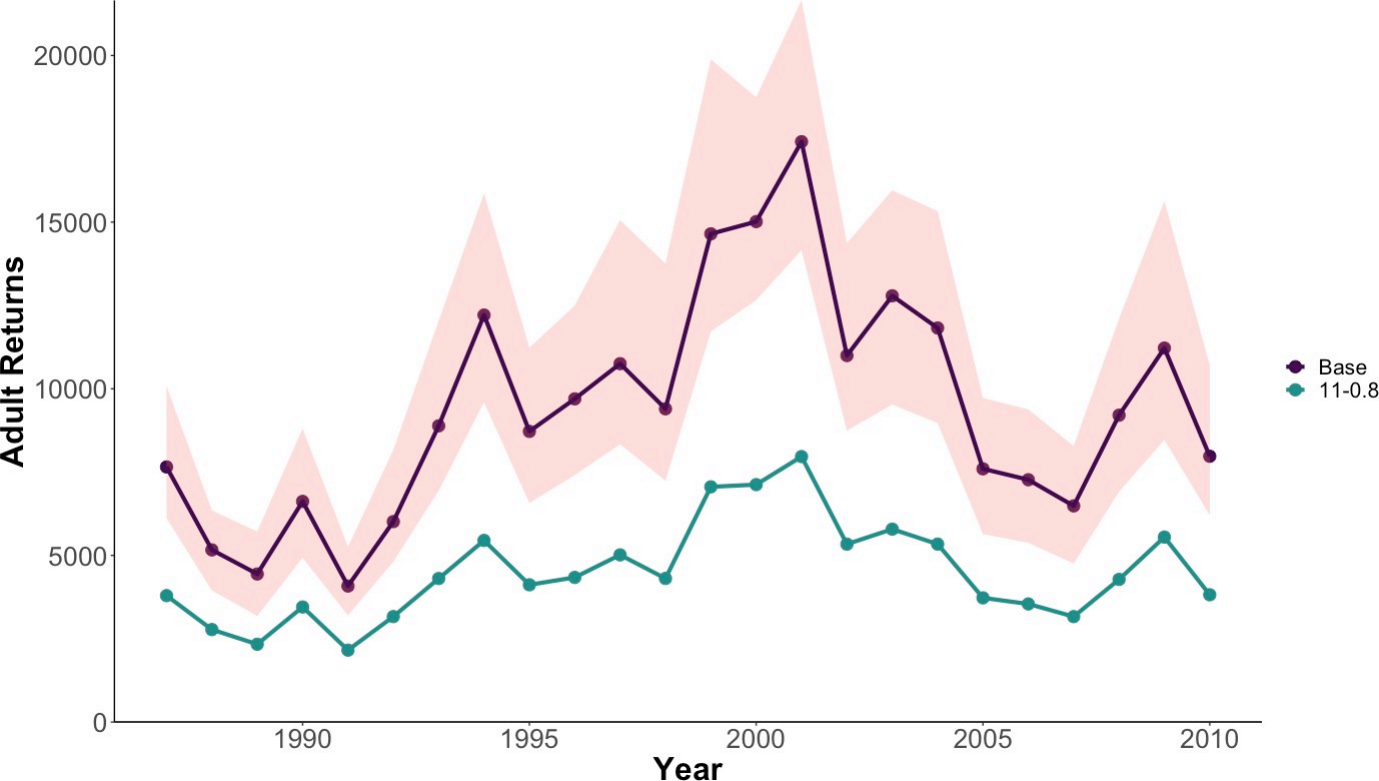
Simulated abundance of adult Chinook salmon returns in the Salcha River indicator stock plotted with (scenario 11–0.8) and without (base) additional mortality. Note how the scenario causes the adult return to fall outside of the 95% Bayesian credible interval (shaded area) of the original model fit.

The simulated late-stage mortality scenarios that most closely replicated observations of shifted age structure concentrated mortality on ocean-age 3 Chinook salmon. These scenarios generated an increase in the proportion of ocean-age 2 returns while not driving the ocean-age 4 returns to unrealistically low levels. Of the 143 scenarios, 90 of them were more likely than the scenarios with no additional mortality (log-likelihood > -170; Table A in S3 File). The top 10 scenarios all selectively removed ocean-age 3 fish and were generated using selectivity suites 8–12 (Fig 2). The age structure and log-likelihood for all scenarios are included in Table A in S3 File. The scenario that produced the final age structure that most closely matched the observed age structure from the Salcha River was 11–0.8 (log-likelihood = -124.97; Table A in S3 File), which divided additional mortality between ocean-age 3 and 4 fish at proportions of 0.9 and 0.1, respectively. This scenario translated to median additional mortality rates of 43% at ocean-age 3 and 6.8% ocean-age 4 on fish entering their third and fourth years at sea. The expected age composition of Chinook salmon returning to the Salcha River under this scenario was: 2.9% ocean-age 1, 17% ocean-age 2, 40% ocean-age 3, 36% ocean-age 4, and 3.8% ocean-age 5. Scenario 11–0.8 resulted in a predicted decrease in adult returns below the lower bound of the 95% Bayesian credible interval from the estimation model fit (Fig 3; [13]). Selectivity suite eight had the greatest cumulative log-likelihood (Fig 2), which distributed 16%, 26%, 5%, and 53% of additional mortality on ocean-ages 2, 3, 4, and 5, respectively.

After using multinomial regression to smooth time series, the scenario that produced the final age structure that most closely matched the age structure for the smoothed 2006–2010 age proportions from the Salcha River was 10–0.6 (log-likelihood = -40.486), which removed only ocean-age 3 fish at an intensity of *I* = 0.6. This scenario translates to a median additional mortality rate of 38% on fish entering their third year at sea. The expected age composition of Chinook salmon returning to the Salcha River under scenario 10–0.6 was: 2.5% ocean-age 1, 15% ocean-age 2, 39% ocean-age 3, 39% ocean-age 4, and 4.1% ocean-age 5. Suite eight still had the greatest cumulative log-likelihood over all intensity levels (Fig B in S2 File).

## Discussion

Our simulations of additional late-stage marine mortality were able to replicate observed age structure truncation of the Salcha River Chinook salmon population. Specifically, a selective scenario that added an additional 43% mortality during the third year at sea and 6.8% during the fourth year best matched the observed trends in age structure compared to a scenario without additional late-stage mortality. All of the most likely scenarios concentrated late-stage mortality during the third year at sea to best replicate the observed age structure in adult returns. As such, the third year at sea may be an underappreciated bottleneck and another critical period in the life of Chinook salmon. Regardless of the specific agents or mechanisms of mortality, the resulting effects could alter the age and possibly sex structure on the spawning grounds and result in further decreases in productivity. Given the imperiled nature of many of these populations, a critical reexamination of possible drivers or agents of mortality after the first year at sea is needed to gain a better understanding of demographic dynamics.

Some potential and non-mutually exclusive causes of marine mortality include changing environmental conditions that affect prey fields, size-selective harvest in directed fisheries, competition with hatchery-derived Pacific salmon, bycatch in marine fisheries and top down forcing by apex predators [30]. Recently emerging evidence suggests that predation is a stronger driver of late–stage marine mortality than selective human harvest and is a primary agent of changes in the age structure of Chinook salmon [31]. Under the assumption that predation is a strong driver of late-stage mortality of Chinook salmon, our top simulation scenarios suggest that the reduction in abundance of the oldest fish on the spawning grounds is not due to predation during those latest ocean years (e.g., ocean-ages 4 and 5), but due to a lack of survival to these ages, particularly through ocean-age 3. We hypothesize that at ocean-age 3 individuals become larger than the average individuals of other prey species, including other highly abundant salmonids (60 cm fork length and greater) [32] and thus may become more conspicuous to visual predators that may select for Pacific salmon. Prior to the third year at sea, these abundant Pacific salmon species such as pink salmon (*O*. *gorbuscha*), chum salmon (*O*. *keta*), and sockeye salmon (*O*. *nerka*) [33] may serve as a predation buffer to growing Chinook salmon, reducing the individual risk of being consumed by a predator, but thereafter individuals may be vulnerable to hyperpredation pressure.

Recent studies have explored the potential impact of recovering marine mammal populations on their Chinook salmon prey [31, 34] and drawn associations between killer whale (*Orcinus orca*) consumption and declines in body size of Chinook salmon across the North Pacific [31]. While killer whales and Chinook salmon are undoubtedly ecologically connected in the coastal waters of British Columbia and Washington State through selective foraging [35], evidence of a killer whale diet preference for Chinook salmon in much of its range, including the Western Pacific Ocean and Bering Sea does not exist. In contrast, direct evidence through satellite tagging of large Chinook salmon has documented substantial predation on ocean age-3 individuals by salmon sharks (*Lamna ditropis*) [36]. Combined, these observations suggest that apex predators frequently consume Chinook salmon, and therefore could act as ecological agents of changing demographics of this species. The corroboration of our simulation exercise and multiple *in situ* predation observations strengthens the inference that Chinook salmon could be facing relatively intense and/or selective mortality in the ocean that could contribute to the change in observed age structure.

Despite proposing a variety of scenarios, the level of late-stage marine mortality necessary to reproduce the observed shift in the age structure did not reproduce estimated abundance. This could be due to an aspect of Chinook salmon population biology that was not included in the model, specifically the heritability of age-at-maturity and how that age can respond to selective pressure. We used a time-invariant probability of maturation to test the numeric removal of individuals alone to aid in the comparison of additional mortality scenarios and because we would not expect the probability of maturation to change significantly over the study period due to evolution. Given a smaller proportion of older-maturing individuals returning to spawn (i.e., ocean-age 4), the lifetime reproductive success of individuals exhibiting this phenotype would be reduced. This would select for earlier maturing individuals, which ultimately could result in a genetic change in the maturation schedule of these populations, to the extent that age at maturity is a heritable trait. Earlier maturation would require the removal of fewer individuals in this simulation framework to observe the same shift in age structure.

Recent work on western Alaskan Chinook salmon using growth histories derived from scale analysis provides evidence that the age threshold for maturation has indeed declined in recent decades [37]. Additionally, simulation work including the heritability of maturation showed that Chinook salmon that experience selective exploitation can experience reductions in the size and age of maturation on the scale of decades [38]. Given the observed decline in maturation threshold, the history of exploitation of this species, our results, and the limitations of the model structure we used, we believe that selective mortality through predation may be playing a part in shaping age structure through sheer removal of individuals. However, observed changes are likely due to a combination of mortality and evolutionary changes in the timing of maturation and additional work is needed to understand the nonlinear effects of these ecological and evolutionary processes.

It is implausible that all observed changes in Chinook salmon demographics are attributable to one agent of selection such as predation. Many of the challenges that Chinook salmon populations face apply selective pressure in the same direction and could exert a cumulative evolutionary effect. Chinook salmon have long been subject to harvest and although harvest rates have been variable through this species’ range and have declined in some areas, harvest is still a mortality source during the late marine stage. Though the impact of bycatch at sea on its own is estimated to be minimal and not very selective [13, 39], bycatch is another source of marine mortality for Chinook salmon after their first year at sea. Additionally, favorable ocean conditions since the 1976/1977 regime shift and hatchery propagation of Pacific salmon has led to more salmon in the ocean, resulting in apparent density-dependent effects [33]. An increasingly crowded ocean could lead to individual fish risking more predation exposure to procure prey and thus experiencing increased marine mortality [40], coupled with reduced growth opportunity. These factors highlight the alignment of several selective pressures in the ocean, which may be individually insignificant, but could compound to exert directional selection for younger age of maturation in Chinook salmon.

The shift in age structure to younger returning adults likely has a further indirect effect by reducing the intrinsic reproductive potential of Chinook salmon populations. This reduction may result from smaller fish having smaller eggs, reduced fecundity, slower swimming speeds, and reduced ability to dig redds, among other fitness consequences [41]. Further compounding these potential ecological effects is the possibility of increasing the skewed sex ratio of adult returns in this species. Chinook salmon are known to have uneven and sometimes extreme sex ratio bias with more male returns than females [42, 43], despite even numbers of individuals going to sea. Presumably due to increased fitness benefits of size-dependent fecundity, female Chinook salmon mature at a larger size and older ages than males [41] and therefore are exposed to more mortality risk in the marine environment. Some rivers and tributaries have experienced low proportions of female returns, which may be reducing the effective population productivity for a given spawning abundance. These rivers include: the mainstem Yukon (33% female, long-term average 42%) and Gisasa rivers (29.6% female), a tributary to the Yukon River in Alaska [26]; the Tuluksak River (31% female), a tributary to the Kuskokwim River, AK [44]; and the Killey River, AK (28% female), a tributary to the Kenai River [45]. Selective late-stage marine mortality could lead to even lower proportions of female returns, which would lead to decreased population fecundity and further reduced population productivity.

Mortality of Chinook salmon after the first year in the ocean has often been dismissed as unlikely to have demographic consequences. Our work challenges this assumption as additional late-stage marine mortality was found to contribute to the best simulation of observed changes in age structure in an index population. Given these results, the remaining years at sea should not be dismissed as inconsequential. Taken as a whole, our results call for a critical reexamination of a longstanding hypothesis in Pacific salmon ecology that the ocean is a generally safe place after their first year at sea, during which the year class strength is set [12]. We suggest that while year class strength may indeed be determined during the first year at sea, population age structure may be determined by selective agents later in the marine stage. Perhaps not surprising, these effects appear the most pronounced in our study species, Chinook salmon, that rear in the ocean longer than other Pacific salmon. In light of the continued depression on Chinook salmon stocks, a critical re-examination of late stage mortality is needed given its apparent role in determine age structure, which in turn can indirectly act as an additional bottleneck to productivity.

## Supporting information

S1 File (CSV)

S2 File (DOCX)

S3 File (DOCX)
