# Supplementary material for: Can late stage marine mortality explain observed shifts in age structure of Chinook salmon?
Source: PLoS One. 2021 Feb 19;16(2):e0247370. doi: 10.1371/journal.pone.0247370 (PMC7895375; doi:10.1371/journal.pone.0247370)
Supplement: S2 File — (DOCX) [file pone.0247370.s002.docx]

# Supporting information 2: Multinomial Modeling Analysis

To test whether inter-annual variation in the age composition of returning adults could be obscuring changes over time, we compared the simulated and observed time-series after reducing them to smoothed trends using multinomial regression. We fit a multinomial log linear regression of the age composition of adults returning to the Salcha River by return year (*43*) to smooth and identify general trends in the highly variable age structure over time. In multinomial regression, the response variable is $y_{i}$ with $k$ response categories, $i=1:k$, and is the log-odds ratio of a response in category $i=2:k$, relative to category 1. Here, $y_{i}$ is the log-odds ratio of adult returns in an older age class in a given calendar year compared to adults returning after one year at sea, thus our model contains five response categories for ocean ages one through five (*k* = 5), which represent the majority of the Salcha River spawning population. The age structure was predicted by the single continuous predictor year *x*, where $\eta$ is the intercept in response category $i$, and $\beta$is the coefficient on $x$ in response category $i$. The linear model is:

(8) $y_{i}= \eta_{i}+\beta_{i}x$,

$y_{i}\sim Multinomial\left( n; p_{i} \right), i=1,2,\ldots,5$;

where n is the number of adults returning in a given year and $p_{i}$ is the probability of those returns belonging to a given ocean age class one through five. Derived from the definition of log-odds, the probability of a fish returning at a specific age and year (*p_i_(x)*) is given by:

(9) $p_{1}\left( x \right)=\frac{1}{1+\sum_{i=2}^{k} \exp\left[ \eta_{i}+\beta_{i}x \right]},$for ocean-age 1 (the reference age class) and

(10) $p_{i}\left( x \right)=\frac{\exp\left[ \eta_{i}+\beta_{i}x \right]}{1+\sum_{i=2}^{k} \exp\left[ \eta_{i}+\beta_{i}x \right]}, i=2,3,4,5$, for remaining marine stages.

Multinomial regression was performed on the time series that resulted from each simulation scenario and the observed age structure reported by the JTC. The overlapping part of the resulting regressions (2006 – 2010) was then compares using the likelihood method described in Equation 7. Total likelihood for each simulation suite was also calculated.

The observed proportions of ocean-age 1 and 2 Chinook salmon from the Salcha River increased over time, while the ocean-age 3, 4, and 5 fish decreased over time (Figure A). Specifically, when examining the age structure trends after smoothing via multinomial regression, the proportion of ocean-age 2 fish increased by 11% while the proportion of ocean-age 3 fish showed a decrease of 12% over the time period from 2006 to 2016. The age structure of Chinook salmon returning to the Salcha River predicted from the smoothed trends for 2010 was: 0.28% ocean-age 1, 17% ocean-age 2, 35% ocean-age 3, 45 % ocean-age 4, and 1.1% ocean-age 5.


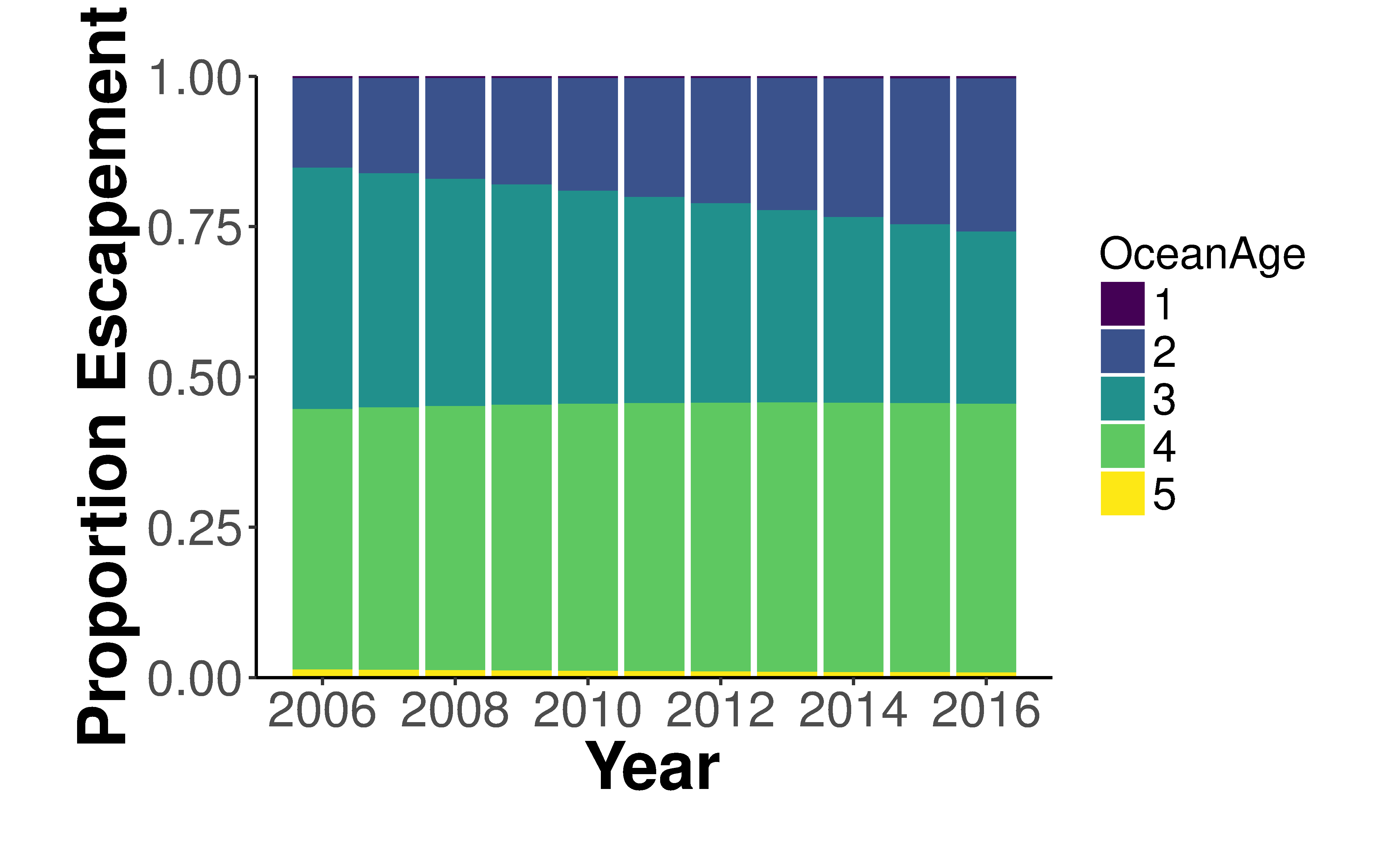


**Figure A:** Trend in proportion of Chinook salmon adult returns to the Salcha River based on the multinomial regression of observed returns from 2006 – 2016 (19,20,29,21–28).

The scenario that produced the final age structure which most closely matched the age structure for the smoothed 2006 – 2010 age proportions from the Salcha River was 10-0.6 (log-likelihood = -40.486), which removed only ocean-age 3 fish at an intensity of $I=0.6$. This scenario translates to a median additional mortality rate of 38% on fish entering their third year at sea. The expected age composition of Chinook salmon returning to the Salcha River under scenario 10-0.6 was: 2.5% ocean-age 1, 15% ocean-age 2, 39% ocean-age 3, 39% ocean-age 4, and 4.1% ocean-age 5. Suite eight still had the greatest cumulative log-likelihood over all intensity levels.

Lewis et al. (7) showed that the proportion of ocean-age 3 fish has increased while the proportion of ocean-age 4 has decreased over time in Yukon River Chinook salmon. However, when examining trends the Salcha River, appears to show nearly a nearly stable proportion of ocean-age 4 fish with a decrease in ocean-age 3. The lack of downward trend in the proportion of ocean-age 4 returns appears to be due to strong returns of that age in 2011, 2012, and 2013 to the Salcha River. The corresponding brood years 2005, 2006, and 2007 did not show large total returns to the mainstem Yukon River (26), however more localized brood year effects could have taken place.

When only the trends in both simulated and observed age structure of returns were considered scenario 10-0.6 was the most likely (Figure B). This scenario concentrates all additional mortality on ocean-age 3, resulting in an additional 38% mortality rate during that year. This result further supports the idea that ocean-age 3 may be an underappreciated survival bottleneck for this species.


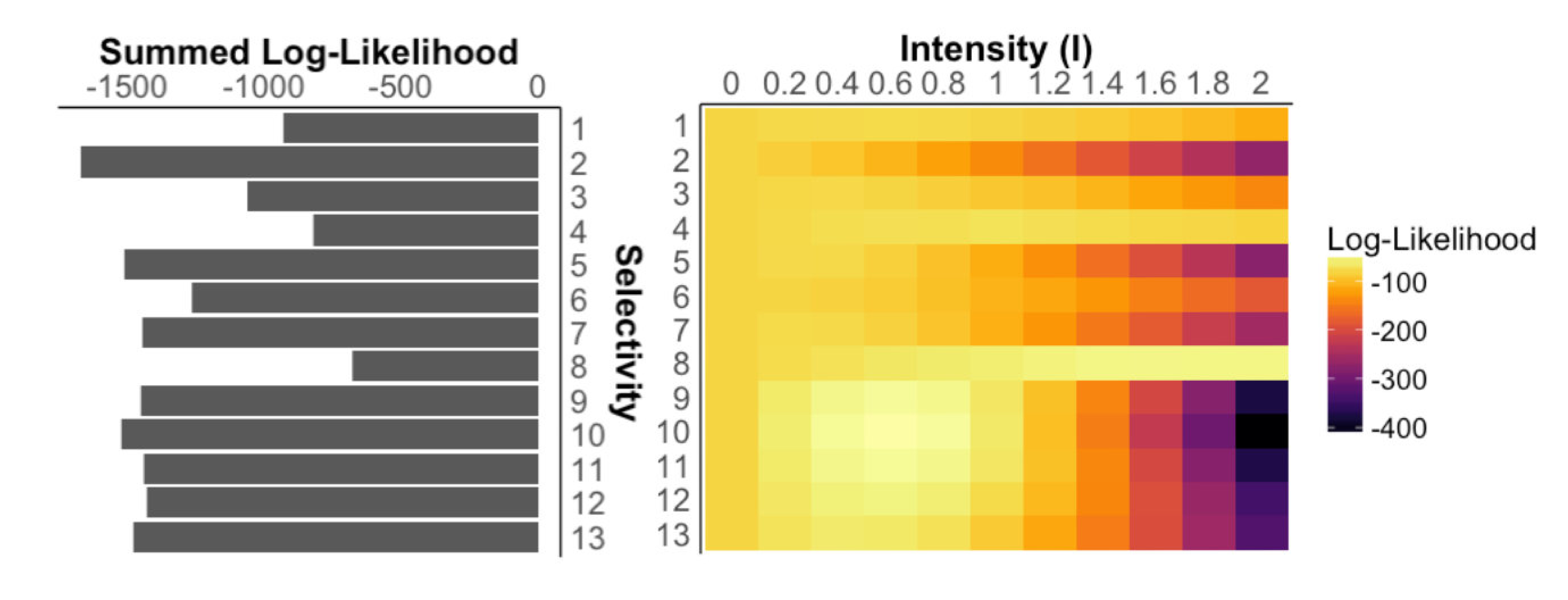


**Figure B:** (Right Panel) Heat map of the log-likelihoods of all scenarios by selectivity suite (*q*) and overall intensity (*I*). (Left Panel) Total likelihood for each selectivity suite integrated over all intensities.
