# Supplementary material for: Can late stage marine mortality explain observed shifts in age structure of Chinook salmon?
Source: PLoS One. 2021 Feb 19;16(2):e0247370. doi: 10.1371/journal.pone.0247370 (PMC7895375; doi:10.1371/journal.pone.0247370)
Supplement: S3 File — (DOCX) [file pone.0247370.s003.docx]

# Supporting information 3: Scenario age structure and log-likelihood table

**Table A:** The simulated age structure and log-likelihood for all 143 scenarios, ordered by log-likelihood of the predicted simulation output given the predicted observed age composition.

|  | Ocean Age | | | | |  |
| --- | --- | --- | --- | --- | --- | --- |
| ID | 1 | 2 | 3 | 4 | 5 | Log-likelihood |
| 11-0.8 | 0.03 | 0.17 | 0.40 | 0.36 | 0.04 | -124.97 |
| 9-0.8 | 0.03 | 0.17 | 0.40 | 0.36 | 0.04 | -125.32 |
| 10-0.8 | 0.03 | 0.18 | 0.38 | 0.37 | 0.04 | -125.59 |
| 12-0.8 | 0.03 | 0.17 | 0.42 | 0.35 | 0.04 | -126.44 |
| 10-0.6 | 0.03 | 0.15 | 0.39 | 0.39 | 0.04 | -126.56 |
| 11-0.6 | 0.02 | 0.15 | 0.41 | 0.38 | 0.04 | -127.14 |
| 9-0.6 | 0.02 | 0.15 | 0.41 | 0.38 | 0.04 | -127.60 |
| 8-2.0 | 0.03 | 0.15 | 0.42 | 0.38 | 0.01 | -129.42 |
| 8-1.8 | 0.03 | 0.14 | 0.42 | 0.38 | 0.02 | -129.55 |
| 12-0.6 | 0.02 | 0.15 | 0.42 | 0.37 | 0.04 | -130.11 |
| 8-1.6 | 0.03 | 0.14 | 0.43 | 0.39 | 0.02 | -132.11 |
| 13-0.8 | 0.03 | 0.16 | 0.44 | 0.34 | 0.04 | -133.63 |
| 13-0.6 | 0.02 | 0.14 | 0.44 | 0.36 | 0.04 | -133.81 |
| 9-0.4 | 0.02 | 0.13 | 0.41 | 0.40 | 0.04 | -135.23 |
| 11-1.0 | 0.03 | 0.20 | 0.38 | 0.34 | 0.04 | -135.29 |
| 10-0.4 | 0.02 | 0.13 | 0.41 | 0.40 | 0.04 | -135.30 |
| 12-1.0 | 0.03 | 0.19 | 0.41 | 0.33 | 0.03 | -135.44 |
| 4-2.0 | 0.04 | 0.14 | 0.45 | 0.34 | 0.03 | -135.70 |
| 8-1.4 | 0.03 | 0.13 | 0.43 | 0.39 | 0.02 | -135.71 |
| 9-1.0 | 0.03 | 0.20 | 0.38 | 0.35 | 0.04 | -135.82 |
| 4-1.8 | 0.04 | 0.14 | 0.45 | 0.35 | 0.03 | -135.97 |
| 11-0.4 | 0.02 | 0.13 | 0.42 | 0.40 | 0.04 | -136.13 |
| 4-1.6 | 0.03 | 0.13 | 0.45 | 0.36 | 0.03 | -137.05 |
| 12-0.4 | 0.02 | 0.12 | 0.42 | 0.39 | 0.04 | -137.87 |
| 10-1.0 | 0.04 | 0.21 | 0.36 | 0.36 | 0.04 | -140.14 |
| 13-1 | 0.03 | 0.19 | 0.43 | 0.32 | 0.03 | -140.25 |
| 13-0.4 | 0.02 | 0.12 | 0.43 | 0.38 | 0.04 | -140.57 |
| 4-1.4 | 0.03 | 0.13 | 0.44 | 0.37 | 0.03 | -140.63 |
| 8-1.2 | 0.03 | 0.13 | 0.43 | 0.40 | 0.02 | -141.00 |
| 4-1.2 | 0.03 | 0.12 | 0.44 | 0.37 | 0.04 | -145.28 |
| 8-1.0 | 0.02 | 0.12 | 0.43 | 0.40 | 0.03 | -145.48 |
| 7-0.6 | 0.02 | 0.13 | 0.46 | 0.36 | 0.04 | -146.42 |
| 1-1.2 | 0.03 | 0.12 | 0.46 | 0.36 | 0.03 | -146.61 |
| 5-0.6 | 0.02 | 0.13 | 0.46 | 0.35 | 0.04 | -147.12 |
| 1-1.6 | 0.03 | 0.14 | 0.47 | 0.34 | 0.03 | -147.65 |
| 4-1.0 | 0.03 | 0.11 | 0.44 | 0.38 | 0.04 | -147.72 |
| 10-0.2 | 0.02 | 0.11 | 0.42 | 0.41 | 0.04 | -148.24 |
| 1-1.4 | 0.03 | 0.13 | 0.46 | 0.35 | 0.03 | -148.62 |
| 8-0.8 | 0.02 | 0.11 | 0.43 | 0.41 | 0.03 | -149.06 |
| 1-1.8 | 0.03 | 0.14 | 0.47 | 0.33 | 0.02 | -149.09 |
| 5-0.4 | 0.02 | 0.11 | 0.45 | 0.38 | 0.04 | -149.18 |
| 7-0.8 | 0.02 | 0.14 | 0.47 | 0.33 | 0.03 | -149.28 |
| 1-1.0 | 0.02 | 0.12 | 0.46 | 0.37 | 0.03 | -149.30 |
| 9-0.2 | 0.02 | 0.11 | 0.42 | 0.41 | 0.04 | -149.61 |
| 1-2.0 | 0.04 | 0.15 | 0.48 | 0.31 | 0.02 | -149.68 |
| 7-0.4 | 0.02 | 0.11 | 0.45 | 0.38 | 0.04 | -149.80 |
| 4-0.8 | 0.02 | 0.11 | 0.44 | 0.39 | 0.04 | -150.72 |
| 5-0.8 | 0.02 | 0.14 | 0.47 | 0.33 | 0.03 | -150.87 |
| 11-0.2 | 0.02 | 0.11 | 0.42 | 0.41 | 0.04 | -151.07 |
| 13-0.2 | 0.02 | 0.11 | 0.43 | 0.40 | 0.04 | -151.81 |
| 1-0.8 | 0.02 | 0.11 | 0.45 | 0.38 | 0.03 | -151.90 |
| 12-0.2 | 0.02 | 0.11 | 0.43 | 0.40 | 0.04 | -151.93 |
| 7-1.0 | 0.03 | 0.15 | 0.48 | 0.31 | 0.03 | -152.89 |
| 8-0.6 | 0.02 | 0.11 | 0.43 | 0.41 | 0.03 | -153.11 |
| 3-1.2 | 0.03 | 0.13 | 0.48 | 0.34 | 0.02 | -153.31 |
| 3-1.0 | 0.02 | 0.12 | 0.47 | 0.36 | 0.03 | -153.74 |
| 3-0.8 | 0.02 | 0.12 | 0.46 | 0.37 | 0.03 | -153.96 |
| 3-0.6 | 0.02 | 0.11 | 0.45 | 0.38 | 0.03 | -154.68 |
| 1-0.6 | 0.02 | 0.11 | 0.45 | 0.39 | 0.04 | -155.13 |
| 5-1.0 | 0.03 | 0.16 | 0.48 | 0.31 | 0.03 | -155.51 |
| 4-0.6 | 0.02 | 0.11 | 0.44 | 0.40 | 0.04 | -155.53 |
| 12-1.2 | 0.04 | 0.22 | 0.40 | 0.31 | 0.03 | -156.40 |
| 3-1.4 | 0.03 | 0.14 | 0.48 | 0.33 | 0.02 | -156.85 |
| 5-0.2 | 0.02 | 0.10 | 0.44 | 0.40 | 0.04 | -157.02 |
| 6-0.6 | 0.02 | 0.11 | 0.46 | 0.37 | 0.03 | -157.11 |
| 7-0.2 | 0.02 | 0.10 | 0.44 | 0.40 | 0.04 | -157.80 |
| 3-0.4 | 0.02 | 0.10 | 0.45 | 0.40 | 0.04 | -158.31 |
| 6-0.8 | 0.02 | 0.12 | 0.48 | 0.36 | 0.03 | -158.79 |
| 8-0.4 | 0.02 | 0.10 | 0.43 | 0.41 | 0.04 | -159.02 |
| 11-1.2 | 0.04 | 0.23 | 0.37 | 0.33 | 0.03 | -159.65 |
| 1-0.4 | 0.02 | 0.10 | 0.44 | 0.40 | 0.04 | -159.65 |
| 3-1.6 | 0.03 | 0.14 | 0.49 | 0.32 | 0.02 | -159.94 |
| 13-1.2 | 0.04 | 0.21 | 0.43 | 0.30 | 0.03 | -160.29 |
| 4-0.4 | 0.02 | 0.10 | 0.43 | 0.41 | 0.04 | -160.41 |
| 6-0.4 | 0.02 | 0.10 | 0.45 | 0.39 | 0.04 | -160.43 |
| 9-1.2 | 0.04 | 0.23 | 0.37 | 0.33 | 0.03 | -160.82 |
| 6-1.0 | 0.02 | 0.12 | 0.49 | 0.34 | 0.03 | -162.13 |
| 4-0.2 | 0.02 | 0.10 | 0.43 | 0.41 | 0.04 | -163.30 |
| 6-0.2 | 0.02 | 0.10 | 0.44 | 0.41 | 0.04 | -163.48 |
| 3-0.2 | 0.02 | 0.10 | 0.44 | 0.41 | 0.04 | -163.63 |
| 1-0.2 | 0.02 | 0.10 | 0.43 | 0.41 | 0.04 | -163.78 |
| 8-0.2 | 0.02 | 0.10 | 0.43 | 0.42 | 0.04 | -164.82 |
| 2-0.4 | 0.02 | 0.10 | 0.47 | 0.38 | 0.03 | -164.94 |
| 7-1.2 | 0.03 | 0.17 | 0.48 | 0.29 | 0.03 | -165.12 |
| 3-1.8 | 0.03 | 0.15 | 0.50 | 0.30 | 0.02 | -165.56 |
| 2-0.2 | 0.02 | 0.10 | 0.45 | 0.40 | 0.04 | -166.67 |
| 6-1.2 | 0.02 | 0.13 | 0.50 | 0.32 | 0.02 | -166.88 |
| 5-1.2 | 0.03 | 0.17 | 0.48 | 0.28 | 0.03 | -167.91 |
| 2-0.6 | 0.02 | 0.11 | 0.48 | 0.36 | 0.03 | -168.77 |
| 10-1.2 | 0.04 | 0.24 | 0.34 | 0.34 | 0.04 | -169.55 |
| 1-0.0 | 0.02 | 0.09 | 0.43 | 0.42 | 0.04 | -171.11 |
| 10-0.0 | 0.02 | 0.09 | 0.43 | 0.42 | 0.04 | -171.11 |
| 11-0.0 | 0.02 | 0.09 | 0.43 | 0.42 | 0.04 | -171.11 |
| 12-0.0 | 0.02 | 0.09 | 0.43 | 0.42 | 0.04 | -171.11 |
| 13-0 | 0.02 | 0.09 | 0.43 | 0.42 | 0.04 | -171.11 |
| 2-0.0 | 0.02 | 0.09 | 0.43 | 0.42 | 0.04 | -171.11 |
| 3-0.0 | 0.02 | 0.09 | 0.43 | 0.42 | 0.04 | -171.11 |
| 4-0.0 | 0.02 | 0.09 | 0.43 | 0.42 | 0.04 | -171.11 |
| 5-0.0 | 0.02 | 0.09 | 0.43 | 0.42 | 0.04 | -171.11 |
| 6-0.0 | 0.02 | 0.09 | 0.43 | 0.42 | 0.04 | -171.11 |
| 7-0.0 | 0.02 | 0.09 | 0.43 | 0.42 | 0.04 | -171.11 |
| 8-0.0 | 0.02 | 0.09 | 0.43 | 0.42 | 0.04 | -171.11 |
| 9-0.0 | 0.02 | 0.09 | 0.43 | 0.42 | 0.04 | -171.11 |
| 6-1.4 | 0.03 | 0.14 | 0.51 | 0.31 | 0.02 | -171.73 |
| 2-0.8 | 0.02 | 0.11 | 0.50 | 0.34 | 0.03 | -174.08 |
| 3-2.0 | 0.03 | 0.16 | 0.50 | 0.29 | 0.02 | -174.49 |
| 7-1.4 | 0.04 | 0.19 | 0.49 | 0.27 | 0.02 | -181.13 |
| 6-1.6 | 0.03 | 0.15 | 0.52 | 0.29 | 0.02 | -183.16 |
| 2-1.0 | 0.02 | 0.12 | 0.52 | 0.33 | 0.02 | -183.90 |
| 5-1.4 | 0.03 | 0.19 | 0.49 | 0.26 | 0.03 | -188.13 |
| 12-1.4 | 0.04 | 0.25 | 0.39 | 0.29 | 0.03 | -189.66 |
| 13-1.4 | 0.04 | 0.24 | 0.42 | 0.27 | 0.03 | -190.21 |
| 6-1.8 | 0.03 | 0.15 | 0.52 | 0.28 | 0.02 | -192.33 |
| 2-1.2 | 0.02 | 0.12 | 0.53 | 0.31 | 0.02 | -198.23 |
| 11-1.4 | 0.04 | 0.27 | 0.35 | 0.30 | 0.03 | -198.50 |
| 9-1.4 | 0.04 | 0.27 | 0.35 | 0.31 | 0.03 | -198.66 |
| 7-1.6 | 0.04 | 0.20 | 0.49 | 0.25 | 0.02 | -204.56 |
| 6-2.0 | 0.03 | 0.16 | 0.53 | 0.26 | 0.01 | -204.82 |
| 10-1.4 | 0.05 | 0.28 | 0.32 | 0.32 | 0.03 | -213.42 |
| 2-1.4 | 0.02 | 0.13 | 0.55 | 0.29 | 0.02 | -213.88 |
| 5-1.6 | 0.04 | 0.21 | 0.49 | 0.24 | 0.02 | -213.97 |
| 13-1.6 | 0.04 | 0.27 | 0.41 | 0.25 | 0.03 | -228.46 |
| 7-1.8 | 0.04 | 0.22 | 0.49 | 0.23 | 0.02 | -231.25 |
| 2-1.6 | 0.02 | 0.13 | 0.56 | 0.27 | 0.01 | -232.10 |
| 12-1.6 | 0.05 | 0.29 | 0.37 | 0.27 | 0.03 | -233.89 |
| 5-1.8 | 0.04 | 0.23 | 0.49 | 0.22 | 0.02 | -242.95 |
| 11-1.6 | 0.05 | 0.30 | 0.34 | 0.28 | 0.03 | -249.97 |
| 9-1.6 | 0.05 | 0.30 | 0.33 | 0.28 | 0.03 | -250.71 |
| 2-1.8 | 0.02 | 0.13 | 0.58 | 0.25 | 0.01 | -252.74 |
| 7-2.0 | 0.05 | 0.24 | 0.49 | 0.21 | 0.02 | -260.79 |
| 10-1.6 | 0.05 | 0.32 | 0.30 | 0.30 | 0.03 | -271.11 |
| 2-2.0 | 0.03 | 0.14 | 0.59 | 0.24 | 0.01 | -275.78 |
| 5-2.0 | 0.05 | 0.25 | 0.49 | 0.20 | 0.02 | -276.90 |
| 13-1.8 | 0.05 | 0.30 | 0.40 | 0.23 | 0.02 | -279.50 |
| 12-1.8 | 0.05 | 0.32 | 0.36 | 0.24 | 0.03 | -289.71 |
| 11-1.8 | 0.06 | 0.34 | 0.32 | 0.26 | 0.03 | -315.59 |
| 9-1.8 | 0.06 | 0.34 | 0.31 | 0.26 | 0.03 | -316.64 |
| 13-2 | 0.06 | 0.33 | 0.38 | 0.21 | 0.02 | -338.43 |
| 10-1.8 | 0.06 | 0.36 | 0.28 | 0.27 | 0.03 | -344.45 |
| 12-2.0 | 0.06 | 0.36 | 0.34 | 0.22 | 0.02 | -357.80 |
| 11-2.0 | 0.06 | 0.38 | 0.29 | 0.24 | 0.02 | -394.61 |
| 9-2.0 | 0.06 | 0.38 | 0.29 | 0.24 | 0.02 | -399.36 |
| 10-2.0 | 0.07 | 0.40 | 0.25 | 0.25 | 0.03 | -442.55 |
